# Supplementary material for: Controls of soil organic matter on soil thermal dynamics in the northern high latitudes
Source: Nat Commun. 2019 Jul 18;10:3172. doi: 10.1038/s41467-019-11103-1 (PMC6639258; doi:10.1038/s41467-019-11103-1)
Supplement: Supplementary file 1 — Supplementary Information [file 41467_2019_11103_MOESM1_ESM.pdf]

Supplementary Information

Controls of soil organic matter on soil thermal dynamics in the northern high latitudes

Zhu et al.

Supplementary Figures

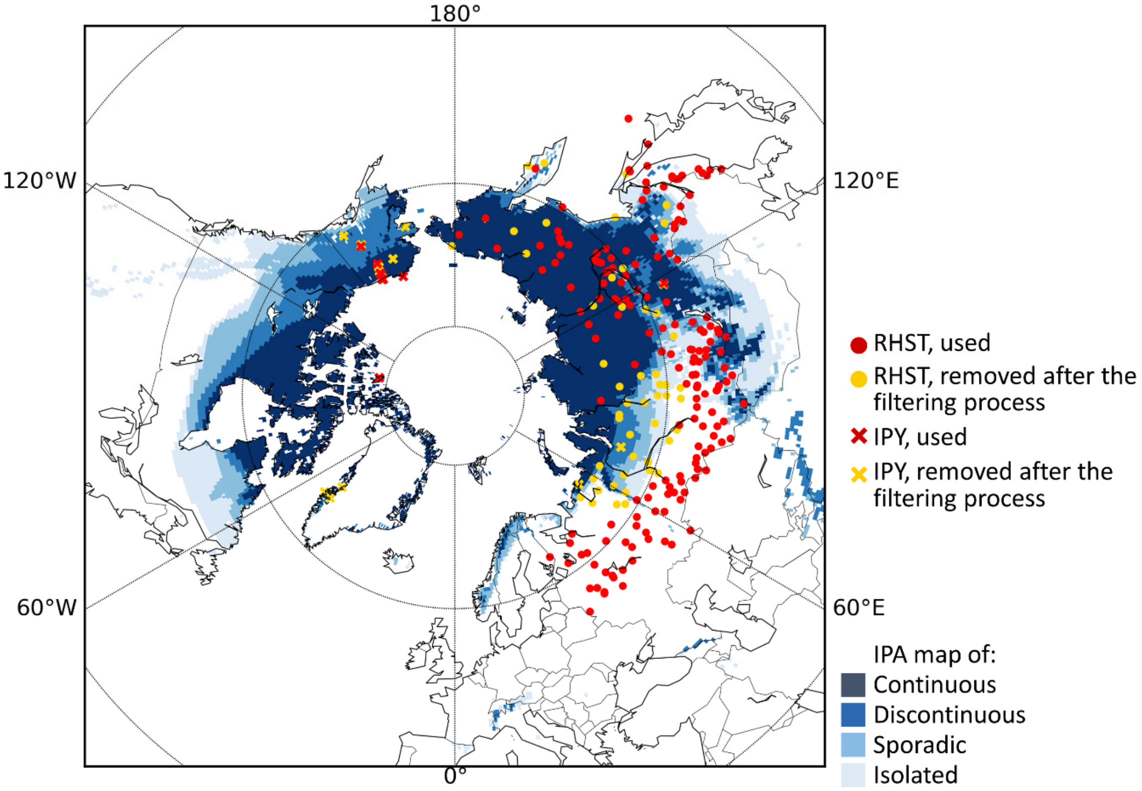

Supplementary Figure 1 | Spatial distribution of the in-situ soil temperature measurements from two datasets: RHST (Russian Historical Soil Temperature Data[1]) shown in circles (219 locations) and IPY (International Polar Year Thermal State of Permafrost[2]) shown in crosses (55 locations). The blue colors indicate the spatial extents of northern permafrost according to the IPA (International Permafrost Association) map[3]. Some observations are excluded (yellow) in the calculation of soil thermal diffusivities, mainly because their monthly temperatures do not conform to a theoretical sine wave, leaving 184 sites (red) for analysis as shown in Fig. 1. See detailed data-filtering process in Methods.

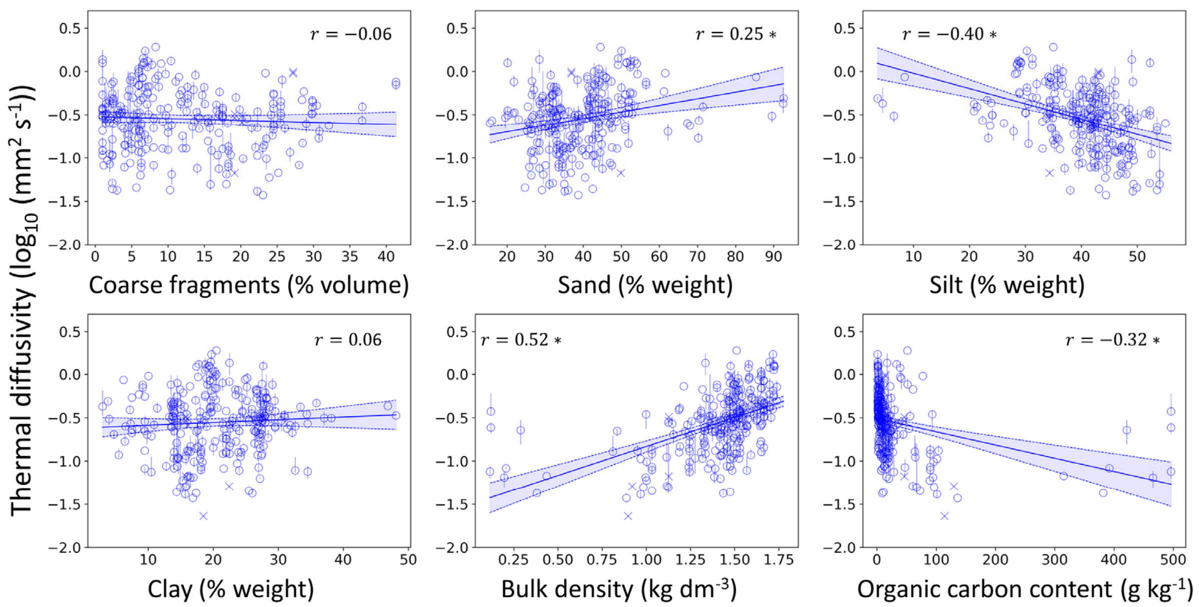

Supplementary Figure 2 | Relationship between soil thermal diffusivity (*D*) derived from depth-specific soil temperature measurements, and soil properties from the WISE database[4]. Each circle represents the median value of *D* over the available years for each site-depth, with 25th – 75th percentiles indicated by the vertical error bars. The Pearson correlation coefficient is labelled in each plot, with an asterix indicating a significant correlation ($p<0.05$). The solid lines represent the linear regression lines, with 95% confidence intervals shown in the dashed lines.

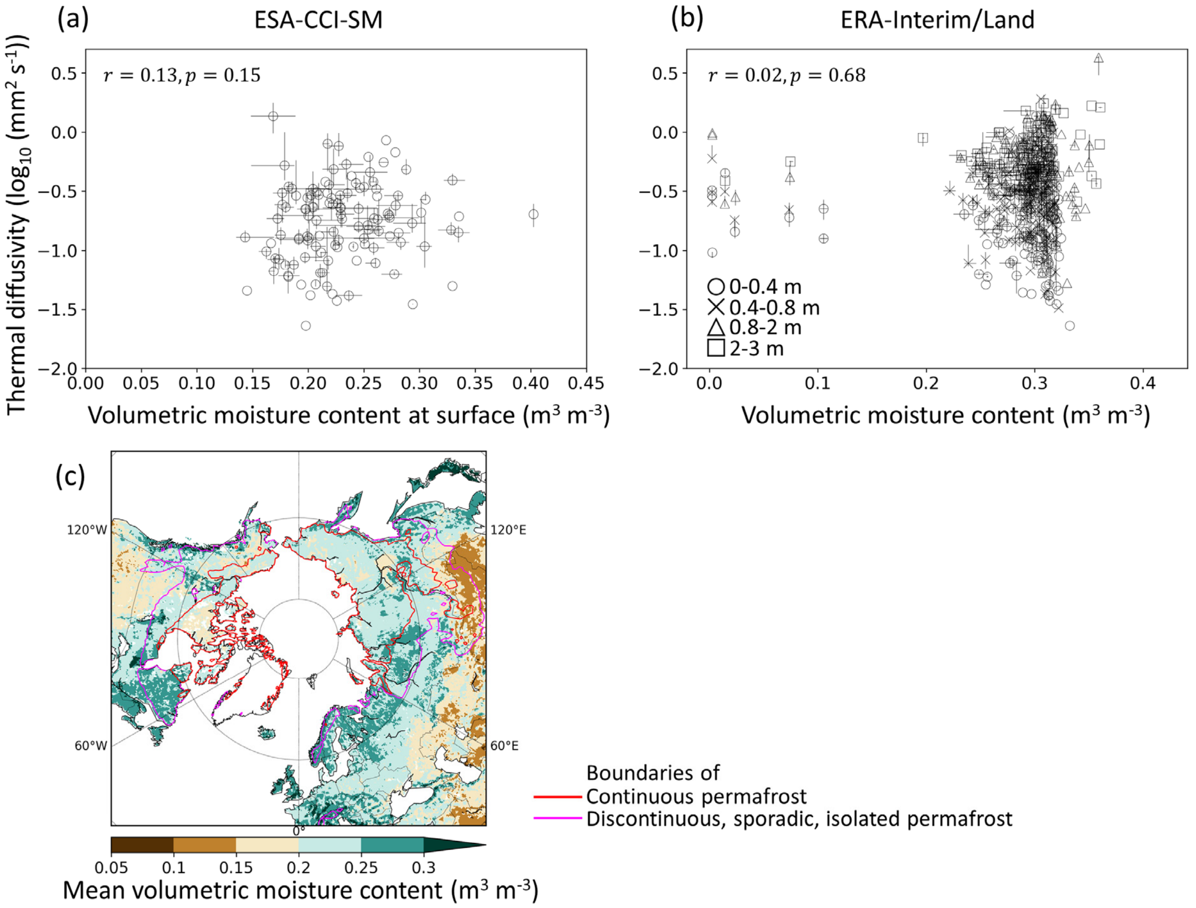

Supplementary Figure 3 | Relationship between soil thermal diffusivity (*D*) and soil moisture from the ESA-CCI-SM v02.2 product[5] **(a)** and the ERA-Interim/Land product[6] **(b)**. ESA-CCI-SM provides satellite-based estimates for surface soil moisture (<10 cm) at 0.25° resolution and at daily time-step but masks out the periods with snow cover or when the soil is frozen[5]. ERA-Interim/Land provides soil moisture at four layers (down to 2.89 m) simulated by a land surface model[6]. For both soil moisture datasets, the mean values over each year are calculated for the site-years where *D* values were available. For ESA-CCI-SM, only the *D* values for the first depth interval (0-0.4 m) were regressed against soil moisture **(a)**; while for ERA-Interim/Land, the soil moisture values at its original soil layers were integrated into the same four depth intervals as *D* values using depth-weighted averages **(b)**. The vertical and horizontal error bars indicate 25th – 75th percentiles among the available years for each site-depth. For both datasets, the Pearson correlation between *D* (before or after log-transformation) and soil moisture is non-significant. **(c)** Annual mean near-surface (<10 cm) soil moisture during 1978-2014 from the ESA-CCI-SM v02.2 product. For each pixel, the years during which too few daily values (<60 days) are available are not included. The red and magenta lines indicate boundaries of northern permafrost according to the IPA map[3].

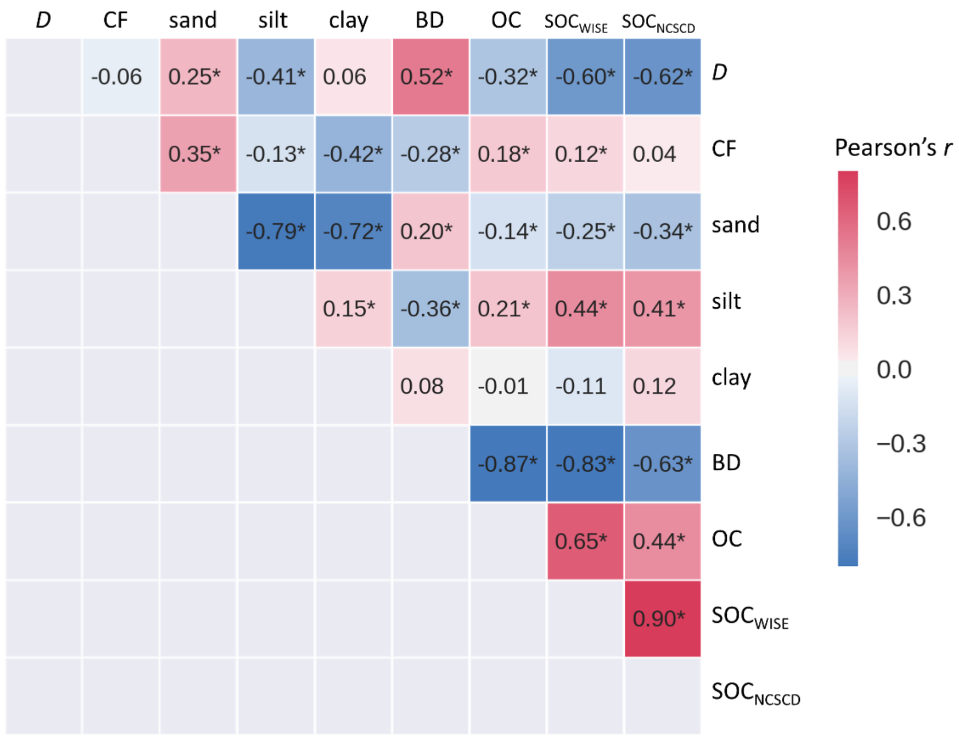

Supplementary Figure 4 | Pearson correlation matrix for *D* and the soil properties. Variable abbreviations: CF: coarse fragment content (% volume). sand, silt, clay: proportions of sand/silt/clay (% weight). BD: bulk density (kg dm^{-3}). OC: organic carbon content of dry weight (g kg^{-1}). These are all from the WISE database[4]. SOC_{WISE} and SOC_{NCSCD}: soil organic carbon density (kg C m^{-3}) from WISE and from NCSCD[7] respectively. All these variables are integrated into the same four depth intervals as the *D* values (0-0.4, 0.4-0.8, 0.8-2, 2-3 m), using depth-weighted means from their original layers in WISE or NCSCD. Significance (*) is evaluated at the 0.05 level.

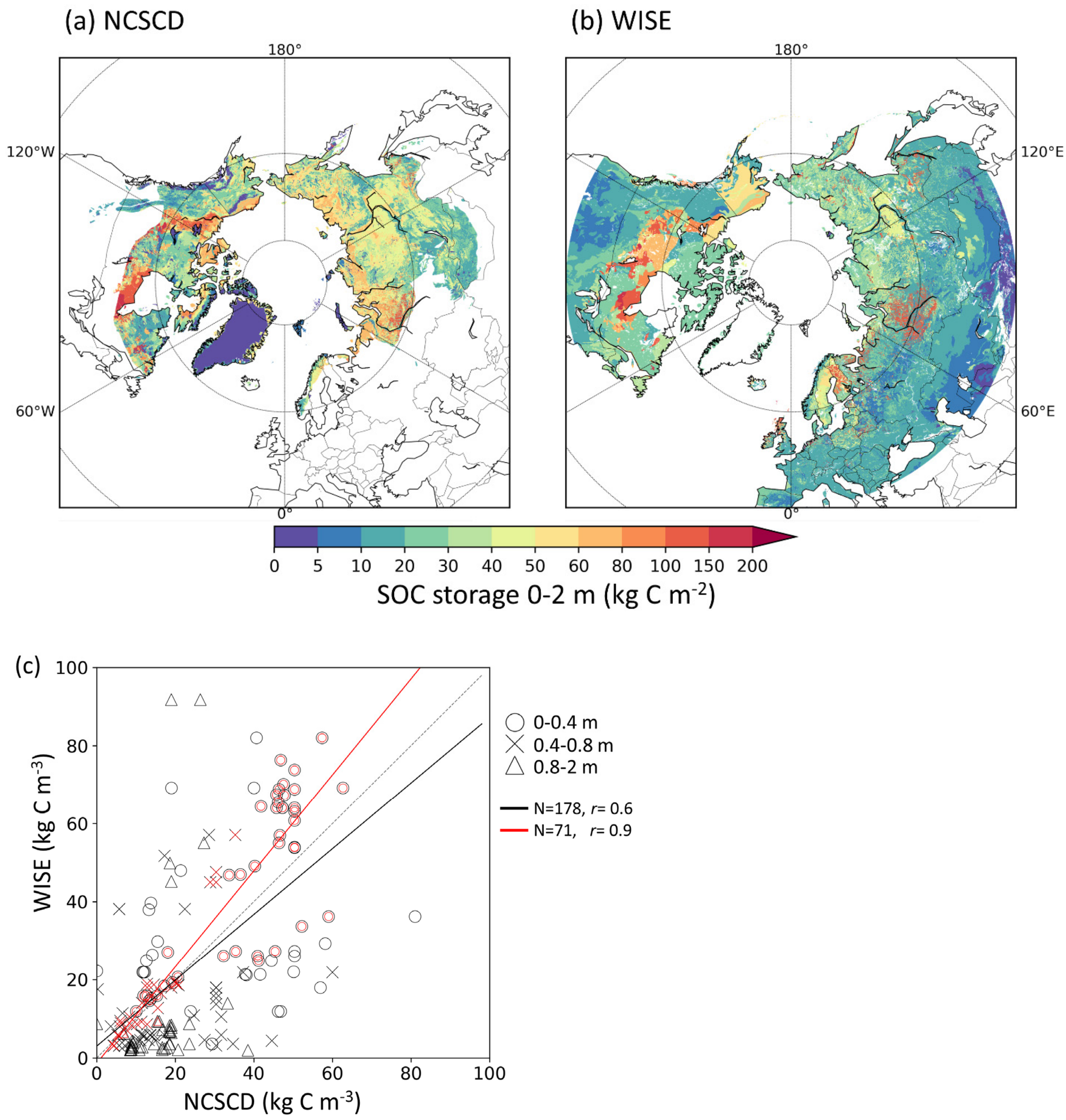

Supplementary Figure 5 | Comparison between the two soil databases used in this study. (**a,b**) Spatial distribution of soil organic carbon stock for 0-2 m depth according to NCSCD and WISE respectively. **(c)** Correlation between SOC densities from NCSCD and WISE for the site-depths where valid *D* values are calculated (see Methods). The red symbols represent a subset of SOC densities whose relative difference between the two databases (calculated as $\left|\frac{SOC_{NCSCD}-SOC_{WISE}}{SOC_{NCSCD}+SOC_{WISE}}\right|$) is less than 25%; while the others are excluded in the analysis of the SOC-*D* relationship as shown in Fig. 1. The solid line represents the linear regression line; the dashed line represents the 1:1 line.

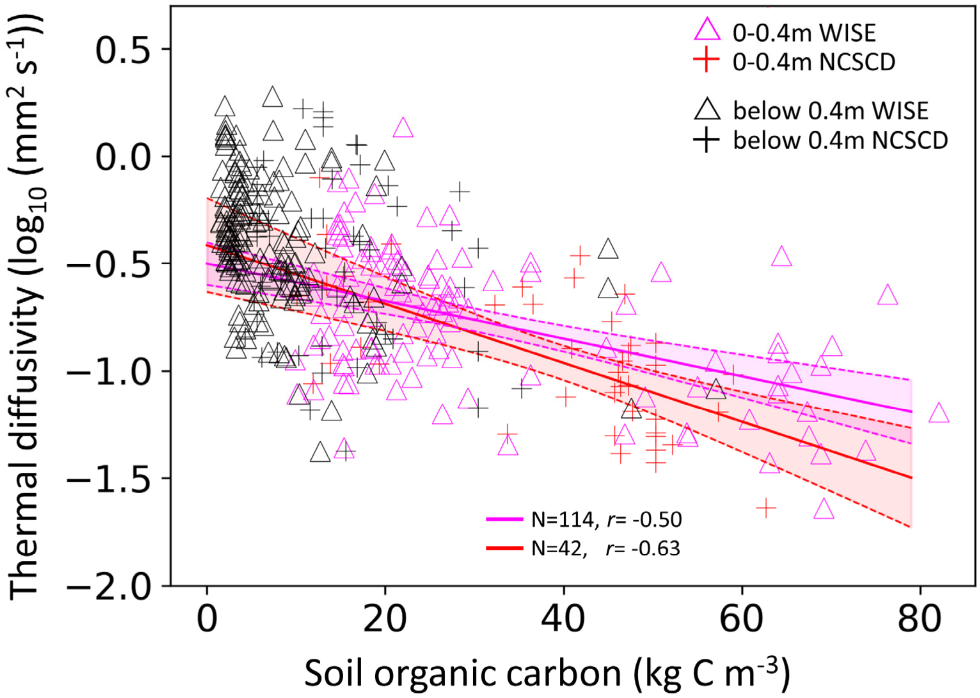

Supplementary Figure 6 | A similar figure as Fig. 1 in the main text but separate the top depth interval (0-0.4 m). The magenta and red solid lines indicate the linear regression lines of $\log_{10}$ (D) versus SOC from WISE (y = -0.009x - 0.50) and from NCSCD (y = -0.014x - 0.42) respectively, with 95% confidence intervals shown in the dashed lines. Both regressions are significant (p<<0.05).

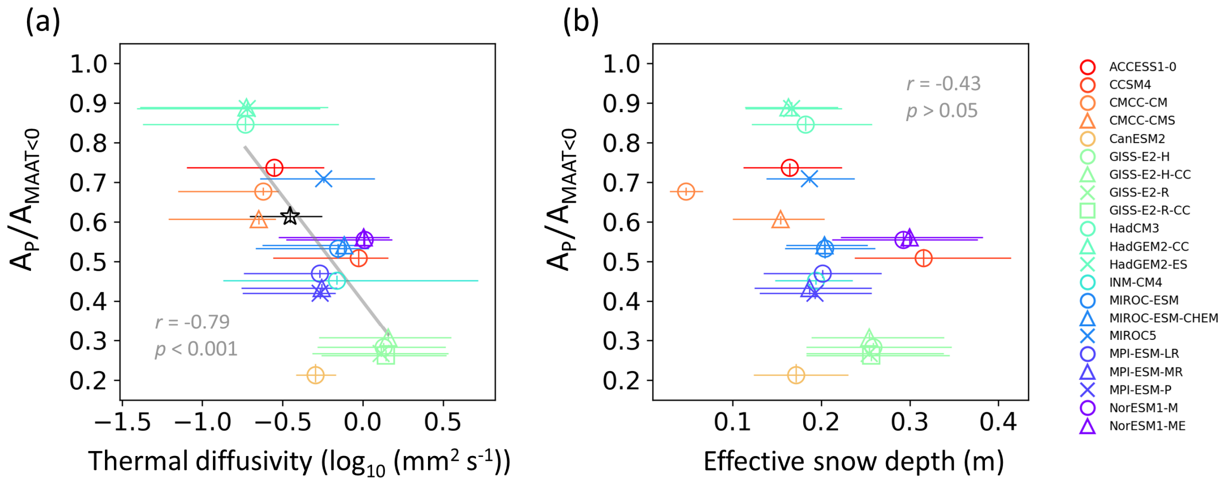

Supplementary Figure 7 | Relationship between $A_p/A_{MAAT<0}$ (the area ratio between near-surface permafrost and regions with MAAT ≤ 0°C) and thermal diffusivity (a) or effective snow depth (b) from CMIP5 models during the period 1961-1990. (a) Same as Fig. 2b except that the thermal diffusivity takes the median of all land pixels whose simulated MAAT ≤ 0°C and monthly soil temperatures conform to theoretical sine waves (SSE<0.34). Less models than Fig. 2b (21 vs. 27) are shown here because the other models did not provide snow depth outputs. The color and shape of each model is the same as in Fig. 2b. The solid grey line represents the linear regression line (y = -0.53x + 0.40). **(b)** The effective snow depth is calculated following ref.[8] (see equation (7)). The median value of all land pixels whose simulated MAAT ≤ 0°C for each model is shown. The error bars indicate 25th – 75th percentiles.

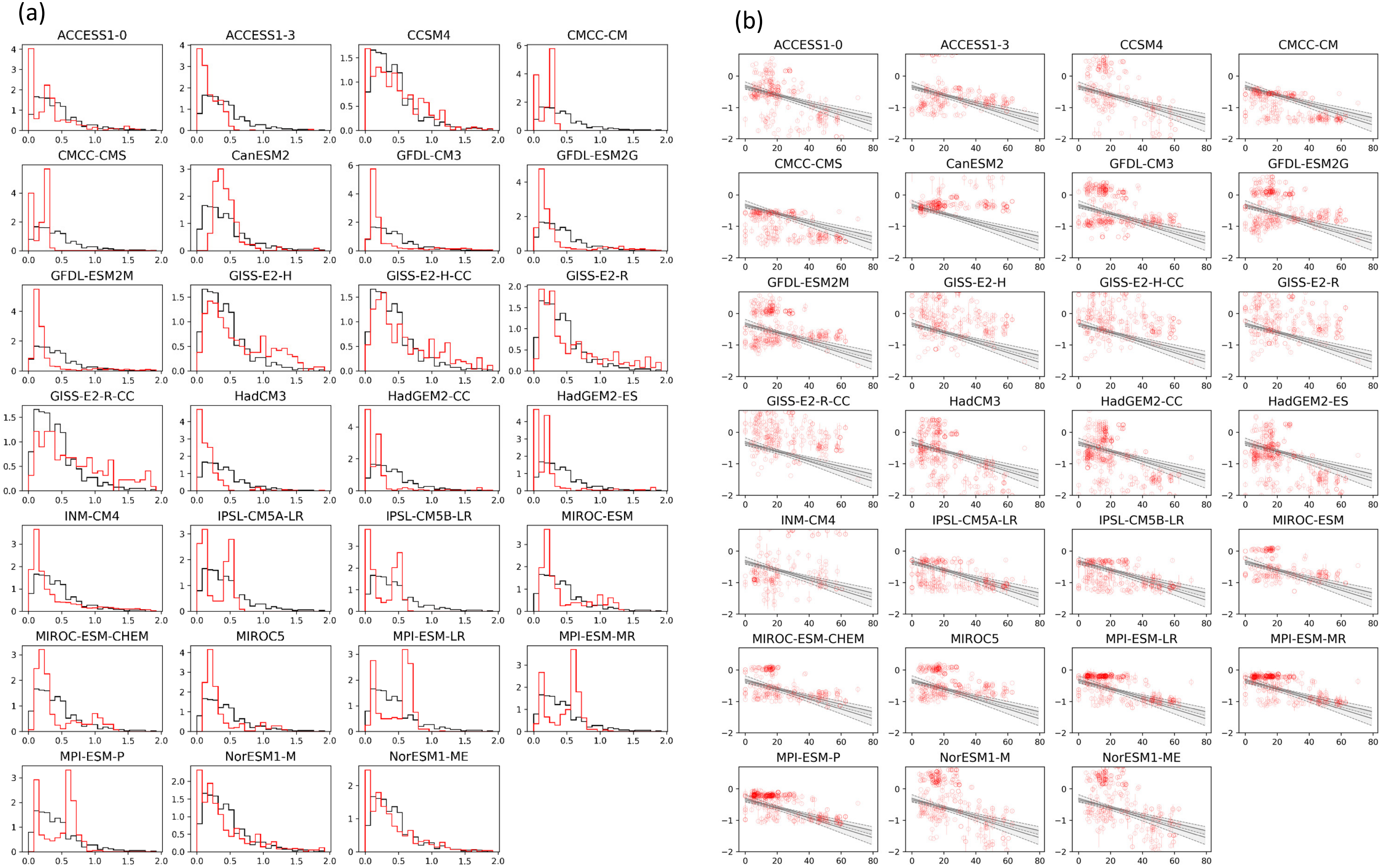

Supplementary Figure 8 | (a) Histogram (normalized frequency) of soil thermal diffusivity (*D*, unit: 10^{-6} m^2 s^{-1}) retrieved from soil temperature profiles from CMIP5 models (in red) for the same locations as the soil temperature observations, compared with the observation-derived *D* values for these sites (in black). **(b)** Relationship between log-transformed *D* by each model and SOC density (kg C m^{-3}, according to NCSCD (for sites in permafrost zones) or WISE (for non-permafrost sites) databases) for the same site locations. The grey lines indicate the empirically-derived relationships (the same as in Fig. 1).

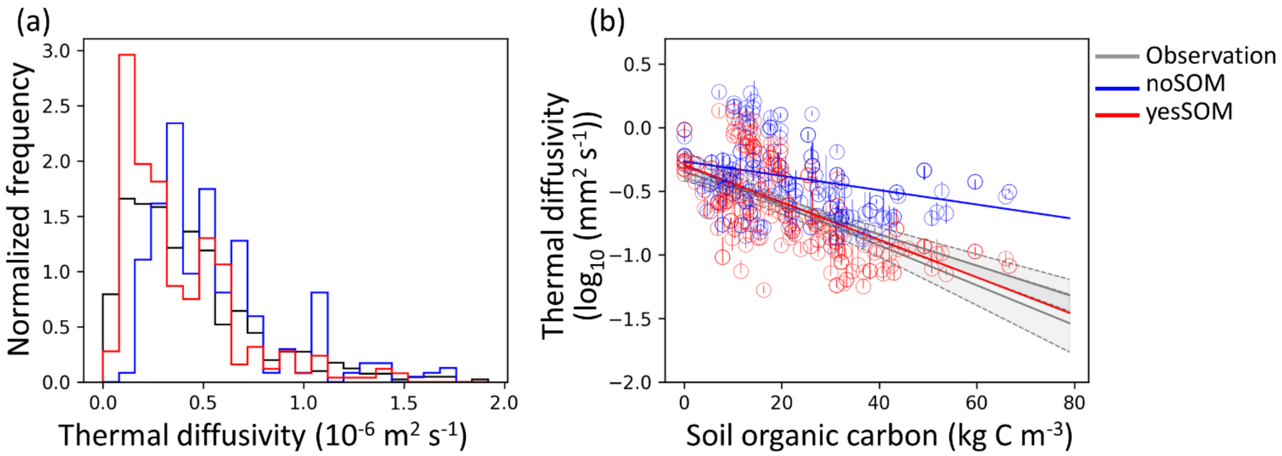

Supplementary Figure 9 | Normalized frequency (a) and regression against SOC (b) of soil thermal diffusivity (*D*) simulated by ORCHIDEE-MICT without (noSOM, in blue) or with (yesSOM, in red) the impact of organic carbon on soil thermal and hydrological properties, for the same locations as the soil temperature observations, compared with the observation-derived *D* values for these sites (in black). In the calculation of *D* values of the model, the same SSE threshold of 0.34 was adopted to exclude the pixel-depth-years whose monthly soil temperature oscillations do not conform to sine waves. In (b), the red and blue lines indicate the linear regression lines for yesSOM and noSOM respectively, and the grey lines indicate the empirically-derived relationships (the same as in Fig. 1).

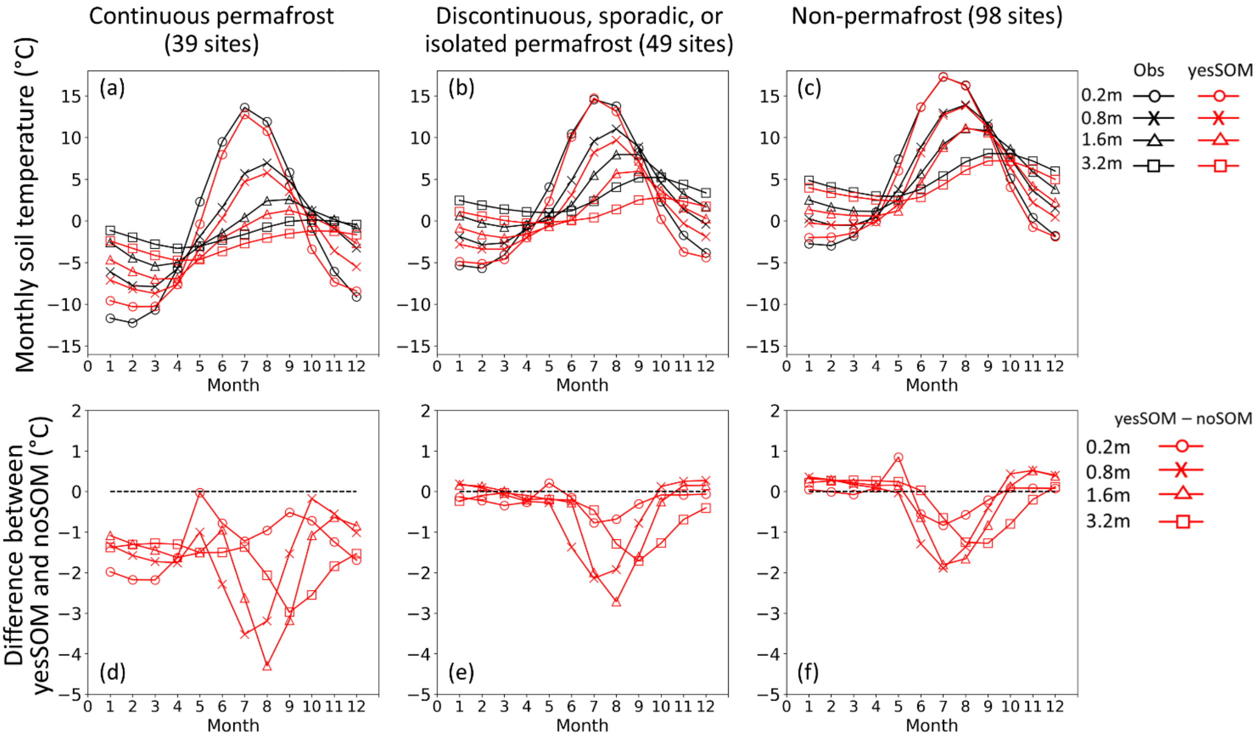

Supplementary Figure 10 | (a-c) Monthly mean soil temperature at four depths averaged over the sites of observations from RHST and IPY datasets (in black), compared to the model results at the same locations considering SOM effect (yesSOM, in red). Since the measurements in IPY dataset have variable layers at different sites, we took the nearest available depth at a site if its difference from the shown four depths is less than 0.2 m. Only the sites with available data for all four depths were used for the averaging. The sites were separated into three regions: continuous permafrost (left panel), discontinuous, sporadic, or isolated permafrost (middle panel), and non-permafrost (right panel). **(d-f)** Difference in modelled soil temperature between yesSOM and noSOM, averaged over the same locations.

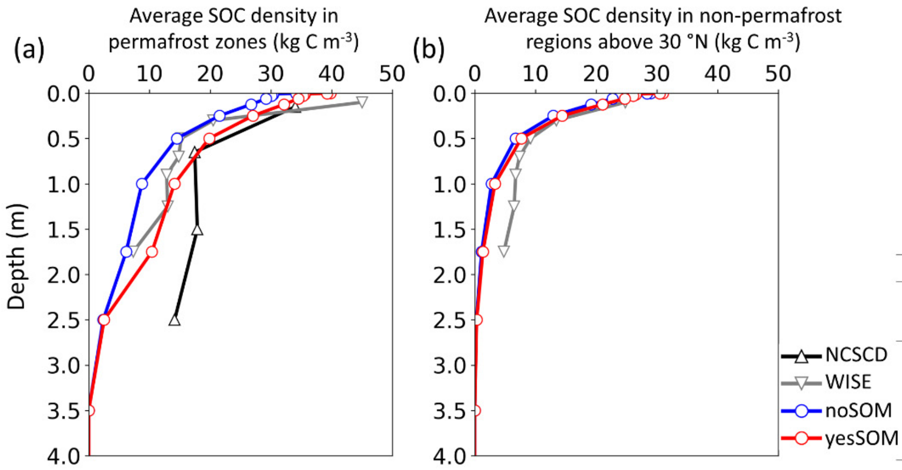

	Total SOC storage (Pg C)					
	Permafrost			Non-permafrost (>30°N)		
	0-1m	1-2m	2-3m	0-1m	1-2m	2-3m
NCSCD	470	355	206	/	/	/
WISE	480	210	/	463	213	/
noSOM	372	164	52	406	82	12
yesSOM	492	271	56	454	99	14

Supplementary Figure 11 | Modelled vertical profiles of soil organic carbon, compared with NCSCD and WISE databases, averaged for the northern permafrost zones according to the IPA map including all the four permafrost categories (total area, 22 million km^2) **(a)**, or for non-permafrost regions above 30°N **(b)**, with total SOC storages listed in the inserted table.

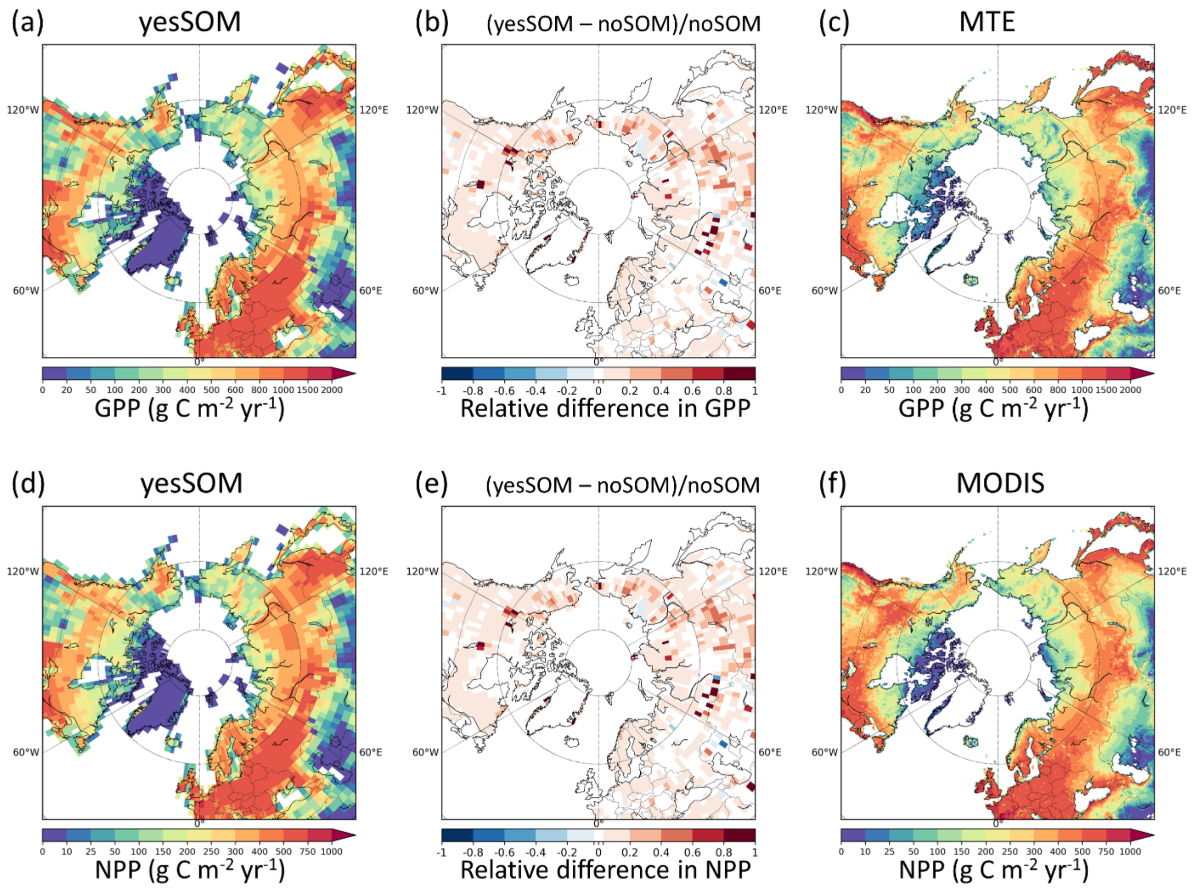

Supplementary Figure 12 | Modelled mean gross and net primary productivity (GPP and NPP) during 1990-2010 from yesSOM **(a,d)**, and the difference between noSOM and yesSOM **(b,e)**. **(c)** Data-driven product Model Ensemble Tree (MTE) GPP[9] averaged for the period 1990-2008. **(f)** Satellite-derived product MODIS NPP[10] averaged for the period 2000-2010.

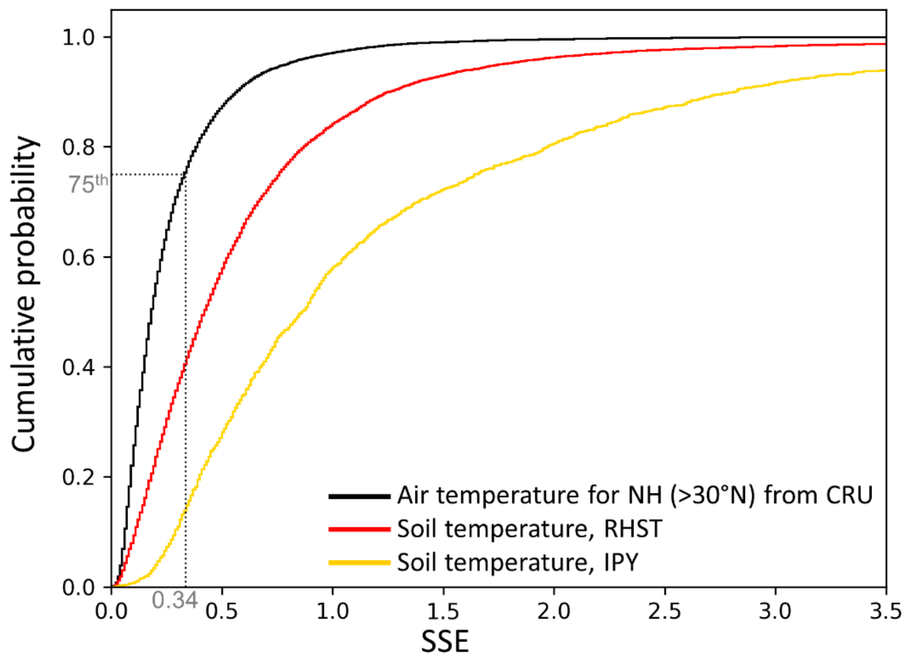

Supplementary Figure 13 | Cumulative probability distributions of SSE (sum of square error, see equation (6)) of monthly air temperature for all grid cells in the northern hemisphere (>30°N) from CRU dataset during 1981-1990 (black), and monthly soil temperature of all site-depth-years from RHST dataset (red) and IPY dataset (yellow). A smaller SSE indicates a higher similarity between the monthly temperature oscillations and a theoretical sine-wave function.

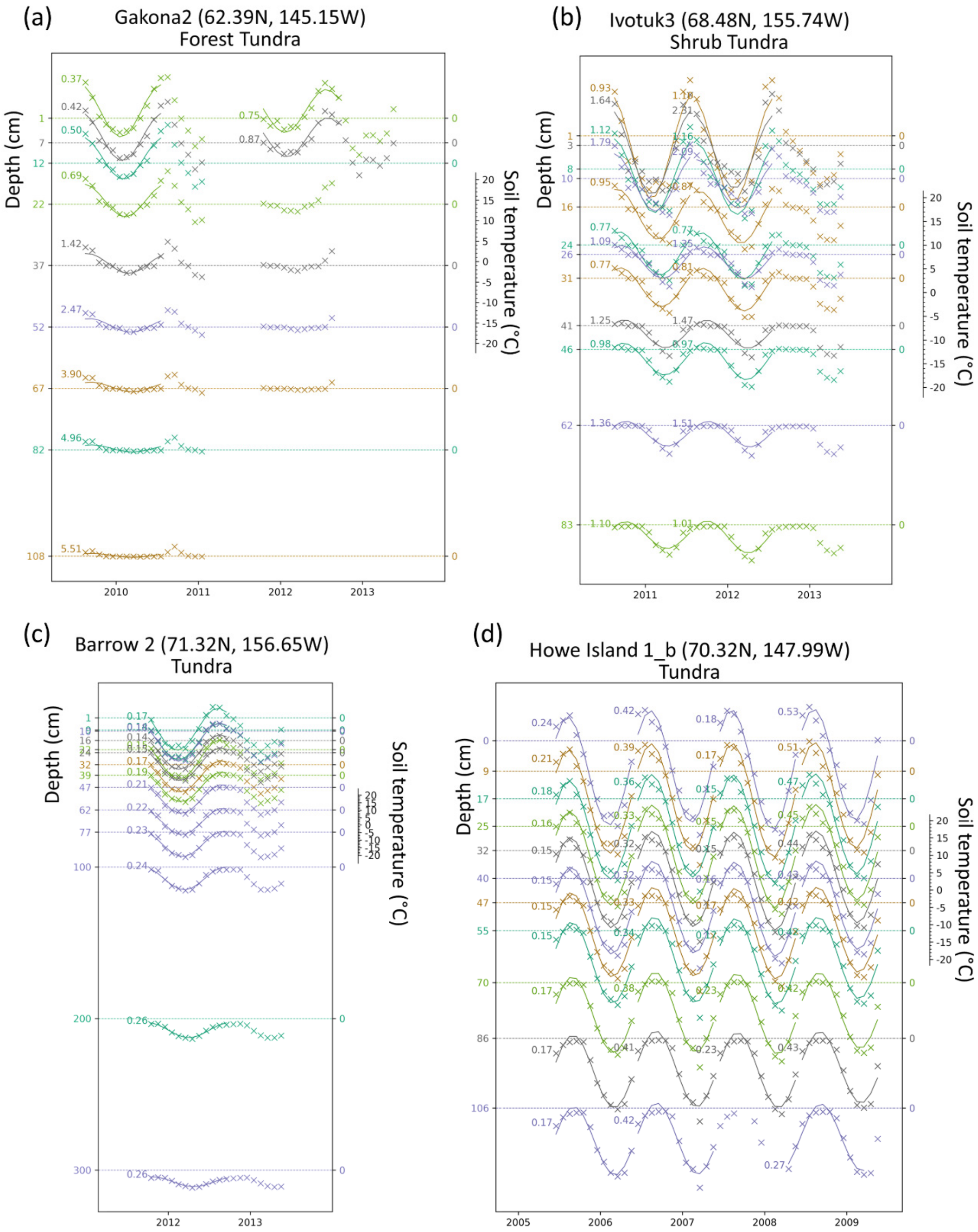

Supplementary Figure 14 | Examples from four sites to illustrate the differences (a,b) or conformity (c,d) between observed monthly soil temperatures and theoretical sine waves. Crosses represent temperature measurements, integrated to monthly means. Solid curves represent the fitted sinusoidal functions based on each consecutive 12-month observations. The horizontal dashed lines represent the depths (left y-axis) or 0 °C temperature (right y-axis). Data on adjacent depths are shown in different colors for legibility. The SSE (sum of square error, see equation (6)) values for each fitted curve are labelled beside the curves. An SSE threshold of 0.34 was chosen to filter the site-depth-year observations such that those with higher SSE values were excluded in the calculation of soil thermal diffusivities (see Methods).

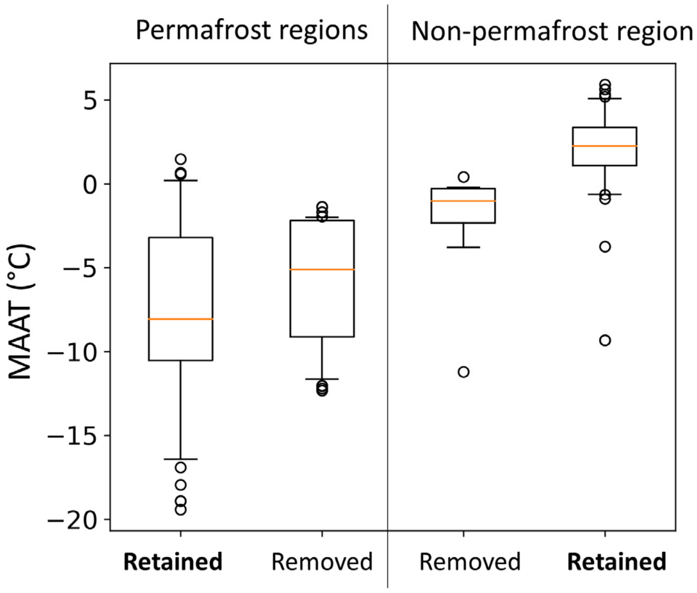

Supplementary Figure 15 | Mean annual air temperature (according to CRU TS v3.24 climate dataset, averaged for 1981-1990) of the retained and removed sites in permafrost (including continuous and discontinuous categories) and non-permafrost regions respectively. The box plots show the median (orange line), the 5th and 95th percentiles (whiskers), and outliers (circles). The difference is significant for both regions (two-sided t-test, $p=0.03$ for permafrost regions, $p<<0.05$ for non-permafrost region).

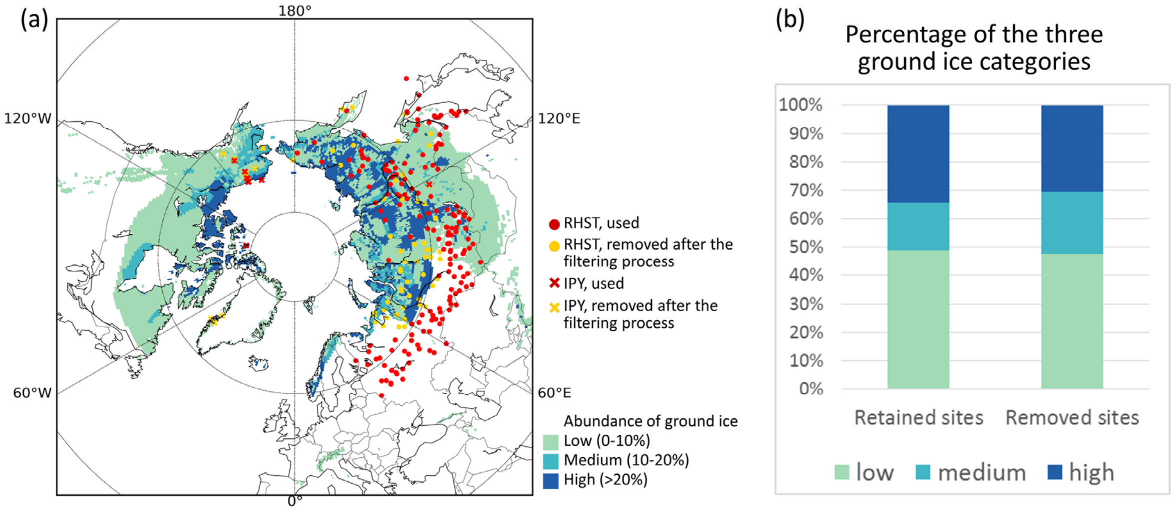

Supplementary Figure 16 | **(a)** Similar to Supplementary Figure 1 except that the base map shows ground ice abundance according to the IPA Map[3]. **(b)** Percentage of the three ground ice abundance categories for the removed and retained sites (in permafrost regions only), showing that the data filtering does not preferentially remove sites with high ground ice contents.

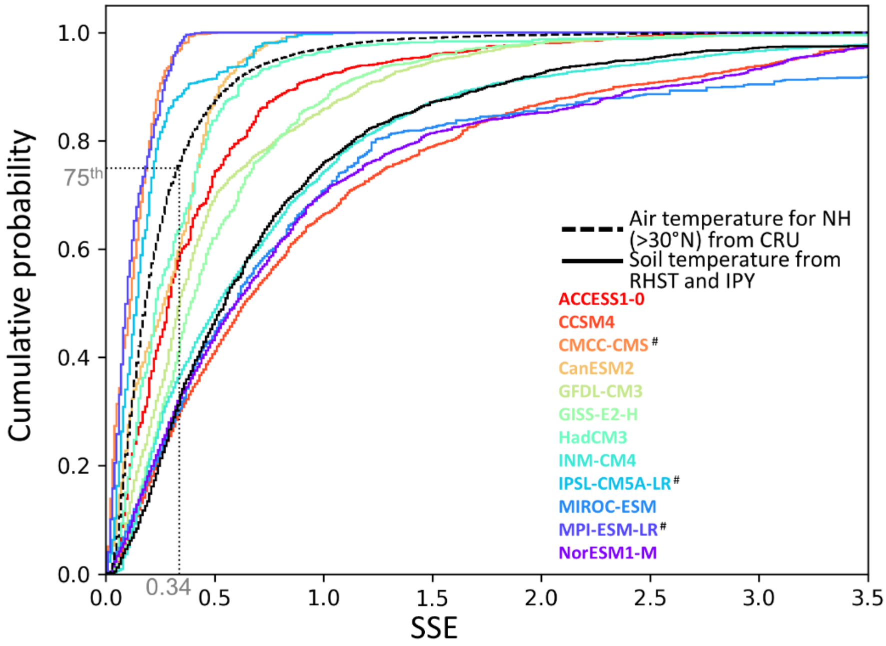

Supplementary Figure 17 | Cumulative probability distributions of SSE (sum of square error, see equation (6)) of monthly air temperature for all grid cells in the northern hemisphere (>30°N) from CRU dataset during 1981-1990 (dashed black), of monthly soil temperature for all site-depth-years from RHST and IPY datasets (solid black), and of simulated monthly soil temperature by CMIP5 models for the period 1981-1990, for all model layers above 3 m at the same RHST and IPY sites. The color of each model is the same as in Fig. 2b. Models of the same family have similar curves, thus only one model for each family is shown for legibility. A smaller SSE indicates a higher similarity between the monthly temperature oscillations and a theoretical sine-wave function. Three model families (marked with #) do not consider the latent heat induced by water phase changes[11], thus have smaller SSE values in general compared to other models.

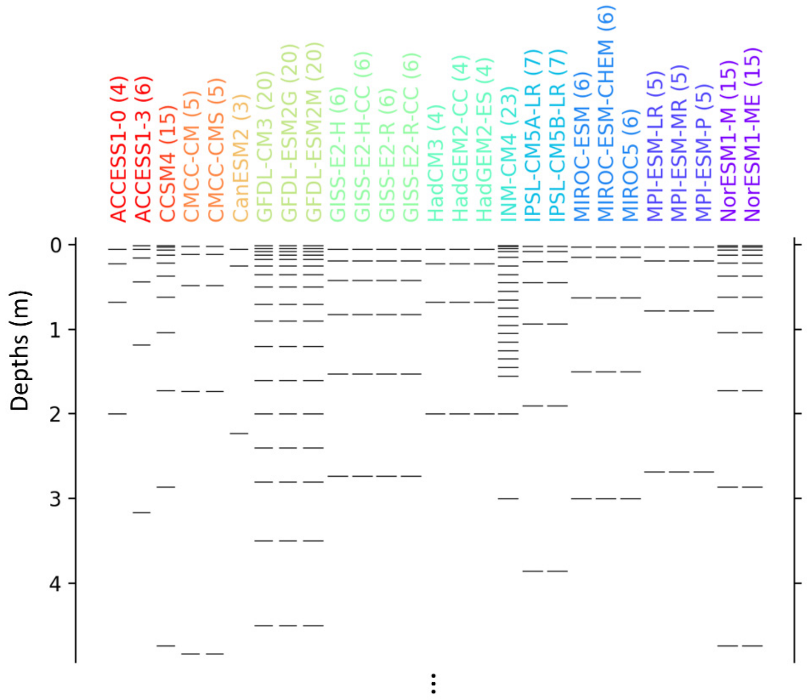

Supplementary Figure 18 | Vertical discretization (above 5 m) for soil temperatures in the CMIP5 models analysed in this study. The number in parenthesis indicates the total number of soil layers in each model.

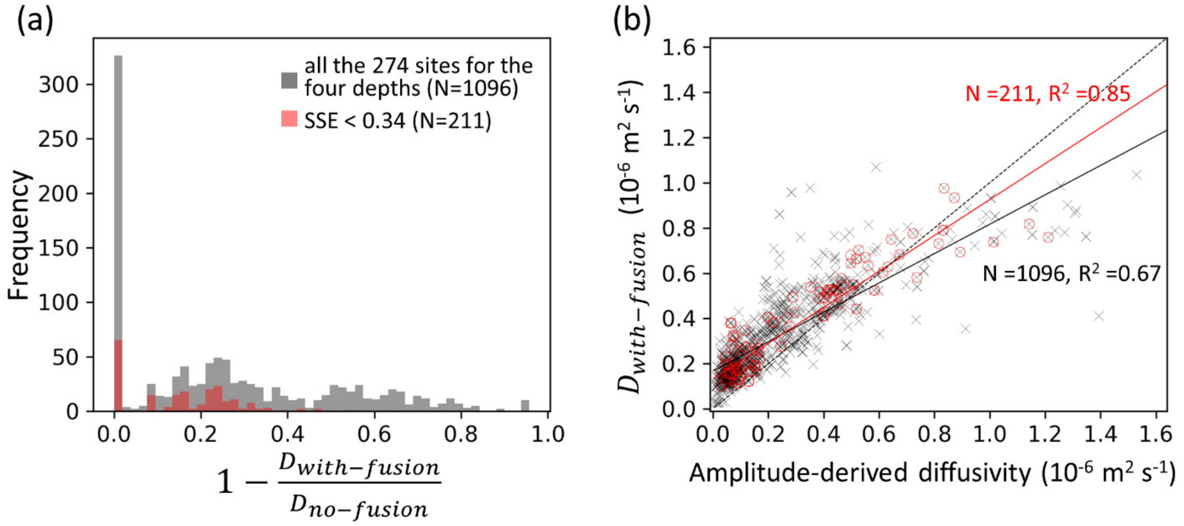

Supplementary Figure 19 | (a) Frequency of the relative difference between $D_{with\text{-}fusion}$ and $D_{no\text{-}fusion}$ (see equation (9)) simulated by ORCHIDEE-MICT (the yesSOM run) at the RHST and IPY sites averaged for the years 1981-1990 (in black), and a subset of them whose SSE of modelled soil temperature is smaller than 0.34 (in red). Diffusivity values for the model's original soil layers were integrated into the four depth intervals: 0-0.4, 0.4-0.8, 0.8-2, and 2-3 m. A higher difference indicates a larger impact of the latent heat induced by soil freezing/thawing on the apparent thermal diffusivity over a year. **(b)** Comparison between the temperature amplitude-derived diffusivity (D) and $D_{with\text{-}fusion}$, the latter representing the values used in the Fourier's equation to solve for soil temperatures in the model. The red circles indicate a subset of the black crosses whose SSE is smaller than 0.34. The solid lines represent the linear regression lines (black: $y = 0.65x + 0.17$; red: $y = 0.80x + 0.13$). The dashed line represents the 1:1 line.

Supplementary Table 1. Partial correlations between *D* and SOC density, controlled for other soil properties separately and all combined. CF: coarse fragment content (% volume). sand, silt, clay: proportions of sand/silt/clay (% weight). BD: bulk density (kg dm^{-3}). OC: organic carbon content of dry weight (g kg^{-1}). These are all from the WISE database[4]. SOC_{WISE} and SOC_{NCSCD}: soil organic carbon density (kg C m^{-3}) from WISE and from NCSCD[7] respectively. All these variables are integrated into the same four depth intervals as the *D* values (0-0.4, 0.4-0.8, 0.8-2, 2-3 m), using depth-weighted means from their original layers in WISE or NCSCD. Significance (*) is evaluated at the 0.05 level.

	**CF**	**sand**	**silt**	**clay**	**BD**	**OC**	**All**[a]
**SOC_{WISE}**	-0.59*	-0.56*	-0.50*	-0.60*	-0.35*	-0.55*	-0.23*
**SOC_{NCSCD}**	-0.61*	-0.56*	-0.53*	-0.58*	-0.50*	-0.55*	-0.33*

[a] Since the three variables, sand, silt, and clay, sum up to 100%, they should not appear simultaneously in the controlling variables. So here 'All' means the other three soil properties plus any two of sand/silt/clay.

Supplementary Discussion

Impact of soil moisture on thermal diffusivity

Generally, soil thermal diffusivity (*D*) changes with volumetric water content in a non-monotonic manner due to different rates of changes in thermal conductivity and heat capacity, with larger changes in the dryer range of water content and smaller changes in the wetter range[12]. However, wide scatter in the relationship between *D* and soil moisture has been observed in both in-situ field measurements[13,14] and laboratory experiments[15]. In terms of its general tendency, *D* of sandy soils increases rapidly from very dry state to an intermediate level of saturation (0.1~0.2 m^3 m^{-3}), and remain stable or slightly decrease under higher water contents; whereas for soils of smaller grains, the tendency is similar while the magnitude of changes in *D* is smaller[15]. As such, the relationship between *D* and soil moisture in the field depends on the actual range of soil moisture in that area.

In order to test if soil moisture can explain the spatial variability of *D* among the sites, and considering the lack of direct observations of moisture content in the sub-surface soil, we used two datasets, the satellite-based surface soil moisture product (ESA-CCI-SM[5]) and the depth-specific soil moisture simulated by a land surface model which has four layers of 0-7, 7-28, 28-100 and 100-289 cm (ERA-Interim/Land[6]). Although the satellite product ESA-CCI-SM only represents near-surface moisture typically for the above 5~10 cm [5], if moisture content at the near-surface is highly (spatially) correlated with the deeper soil layers, the near-surface moisture can still be used for the purpose of testing whether moisture content can explain the spatial variation of *D*. We checked it based on the ERA-Interim/Land product: indeed, its annual mean moisture content for the first layer is highly correlated with that for the deeper three layers among the site locations considered in this study (r=0.98~0.99).

Supplementary Figure 3c displays the annual mean surface soil moisture from ESA-CCI-SM, showing a generally higher than 0.2 m^3 m^{-3} of water contents over the northern high latitudes, a level above/near which moisture content has no or only marginal directional impact on *D*. This may explain the non-significant correlation between *D* and soil moisture among the sites (Supplementary Figure 3ab).

Then, regarding the potential impact of future changes in soil moisture on *D*, it is also important to consider the range of moisture content. Future warming-induced permafrost thaw could enhance drainage[16] and reduce moisture content in the overlying active layer from a highly-saturated state to, more likely, an intermediately-saturated state rather than to a very dry state, as one may infer from today's mean moisture content in the cold regions outside continuous permafrost zone (Supplementary Figure 3c). Besides, the analysis[17] based on CMIP5 model projections showed a small relative change (from -1.5% to +4.5%) during 2080-2099 compared to 1980-1999 for most of today's permafrost zones, while larger reductions (<-5%) occurring in the already very humid regions like boreal Europe and Hudson Bay. Therefore, the future change

in soil moisture is not very likely to substantially change *D* on a broad-scale through its direct impact on *D*. Indirect impacts, however, could be important, as the projected drying in today's peatland regions[17] may lead to soil carbon losses[18] and thus increase *D* through the SOC-*D* relationship (Fig. 1).

Link between soil warming and soil organic carbon changes in cold regions

For soils in cold regions that are permanently or seasonally frozen, the effect of low temperature is two-fold: apart from the general principle of enzyme kinetics (commonly described by Q_{10} function or Arrhenius equation), a frozen condition also limits the availability of extra-cellular liquid water and thus reduce the organic substrate concentration at enzymatic reaction site by inhibiting diffusion[19]. As shown in ref.[20], the climatological Q_{10} in cold regions is much higher than the commonly recognized value of 1.5~2; and only when decomposition rate is set zero below 0 ºC (plus considering depth-resolved soil temperatures) in the CLM4.5 land surface model can the model reproduce this pattern.

Similar to the CLM4.5 model[20], ORCHIDEE-MICT in this study also includes an inhibition of SOC decomposition rate below -1 ºC. This could explain the much higher relative increase of SOC stock (yesSOM compared to noSOM) in permafrost regions than in non-permafrost regions (Fig. 3d), which is disproportionate to the changes in soil temperature (Supplementary Figure 10). Note that several land surface models[21–23] have incorporated the impact of organic matter on soil thermal and hydrological properties, but did not report its effect on soil carbon accumulation in these models. Besides, when used to project future changes in permafrost distribution[24], the models still prescribed a static present-day soil carbon map to calculate soil physical properties, which missed the potential changes in *D* induced by SOC changes. A full coupling between soil thermodynamics and the prognostically simulated SOC in these models is thus critically needed, requiring a realistic representation of present-day SOC distribution as a first step.

Supplementary References

1. Zhang, T., Barry, R., Gilichinsky, D., University of Colorado, Russian Academy of Sciences. 2009. Russian Historical Soil Temperature Data. Version 1.0. UCAR/NCAR - Earth Observing Laboratory. https://doi.org/10.5065/D66971QC. Accessed 06 Jun 2017.
2. Romanovsky, V. E., Smith, S. L. & Christiansen, H. H. Permafrost thermal state in the polar northern hemisphere during the international polar year 2007-2009: A synthesis. *Permafr. Periglac. Process.* **21**, 106–116 (2010).
3. Brown, J., Ferrians Jr., O. J., Heginbottom, J. A., and Melnikov, E. S.: Circum-Arctic map of permafrost and ground-ice conditions (Version 2), National Snow and Ice Data Center, Boulder, CO, USA, available at: http://nsidc.org/data/ggd318.html.
4. Batjes, N. H. Harmonized soil property values for broad-scale modelling (WISE30sec) with estimates of global soil carbon stocks. *Geoderma* **269**, 61–68 (2016).
5. Liu, Y. Y. *et al.* Developing an improved soil moisture dataset by blending passive and active microwave satellite-based retrievals. *Hydrol. Earth Syst. Sci.* **15**, 425–436 (2011).
6. Balsamo, G. *et al.* ERA-Interim/Land: a global land surface reanalysis data set. *Hydrol. Earth Syst. Sci.* **19**, 389–407 (2015).
7. Hugelius, G. *et al.* A new data set for estimating organic carbon storage to 3 m depth in soils of the northern circumpolar permafrost region. *Earth Syst. Sci. Data* **5**, 393–402 (2013).
8. Slater, A. G., Lawrence, D. M. & Koven, C. D. Process-level model evaluation: a snow and heat transfer metric. *Cryosph.* **11**, 989–996 (2017).
9. Jung, M. *et al.* Global patterns of land-atmosphere fluxes of carbon dioxide, latent heat, and sensible heat derived from eddy covariance, satellite, and meteorological observations. *J. Geophys. Res.* **116**, G00J07 (2011).
10. NTSG (Numerical Terradynamic Simulation Group): MODIS GPP/NPP Project (MOD17A3), available at: http://www.ntsg. umt.edu/project/mod17.
11. Koven, C. D., Riley, W. J. & Stern, A. Analysis of Permafrost Thermal Dynamics and Response to Climate Change in the CMIP5 Earth System Models. *J. Clim.* **26**, 1877–1900 (2013).
12. Farouki, O. T. *Thermal properties of soils. Cold Regions Research and Engineering Lab Hanover NH* (1981).
13. de Jong van Lier, Q. & Durigon, A. Soil thermal diffusivity estimated from data of soil temperature and single soil component properties. *Rev. Bras. Ciência do Solo* **37**, 106–112 (2013).
14. Sugathan, N., Biju, V. & Renuka, G. Influence of soil moisture content on surface albedo and soil thermal parameters at a tropical station. *J. Earth Syst. Sci.* **123**, 1115–1128 (2014).
15. Arkhangelskaya, T. & Lukyashchenko, K. Estimating soil thermal diffusivity at different water contents from easily available data on soil texture, bulk density, and organic carbon content. *Biosyst. Eng.* **168**, 83–95 (2017).

16. Liljedahl, A. K. *et al.* Pan-Arctic ice-wedge degradation in warming permafrost and its influence on tundra hydrology. *Nat. Geosci.* **9**, 312–318 (2016).

17. Dai, A. Increasing drought under global warming in observations and models. *Nat. Clim. Chang.* **3**, 52 (2012).

18. Turetsky, M. R., Donahue, W. F. & Benscoter, B. W. Experimental drying intensifies burning and carbon losses in a northern peatland. *Nat. Commun.* **2**, 514 (2011).

19. Davidson, E. A. & Janssens, I. A. Temperature sensitivity of soil carbon decomposition and feedbacks to climate change. *Nature* **440**, 165–173 (2006).

20. Koven, C. D., Hugelius, G., Lawrence, D. M. & Wieder, W. R. Higher climatological temperature sensitivity of soil carbon in cold than warm climates. *Nat. Clim. Chang.* **7**, 817–822 (2017).

21. Lawrence, D. M. & Slater, A. G. Incorporating organic soil into a global climate model. *Clim. Dyn.* **30**, 145–160 (2008).

22. Chadburn, S. *et al.* An improved representation of physical permafrost dynamics in the JULES land-surface model. *Geosci. Model Dev.* **8**, 1493–1508 (2015).

23. Decharme, B. *et al.* Impacts of snow and organic soils parameterization on northern Eurasian soil temperature profiles simulated by the ISBA land surface model. *Cryosphere* **10**, 853–877 (2016).

24. Chadburn, S. E. *et al.* Impact of model developments on present and future simulations of permafrost in a global land-surface model. *Cryosph.* **9**, 1505–1521 (2015).
